# Neural responses to a modified Stroop paradigm in patients with complex chronic musculoskeletal pain compared to matched controls: an experimental functional magnetic resonance imaging study

**DOI:** 10.1186/s40359-016-0109-4

**Published:** 2016-02-01

**Authors:** Ann M. Taylor, Ashley D. Harris, Alice Varnava, Rhiannon Phillips, Owen Hughes, Antony R. Wilkes, Judith E. Hall, Richard G. Wise

**Affiliations:** Department of Anaesthetics, Intensive Care and Pain Medicine, Institute of Infection and Immunity, Cardiff University, Cardiff, CF14 4XN Wales UK; Cardiff University Brain Research Imaging Centre (CUBRIC), School of Psychology, Cardiff University, Cardiff, CF10 3AT Wales UK; Russell H. Morgan Department of Radiology and Radiological Science, The Johns Hopkins University, Baltimore, MD USA; F. M. Kirby Center for Functional Brain Imaging, Kennedy Krieger Institute, Baltimore, MD USA; Department of Psychology, Swansea University, Singleton Park, Swansea, SA2 8PP Wales UK; School of Psychology, Cardiff University, Cardiff, CF10 3AT Wales UK; Institute of Primary Care and Public Health, Cardiff University, Cardiff, CF14 4YS Wales UK; Bronllys Pain and Fatigue Management Centre, Powys, Brecon, LD3 0 LU Wales UK

**Keywords:** Neuroimaging, fMRI, Complex chronic pain, Musculoskeletal, Stroop

## Abstract

**Background:**

Chronic musculoskeletal pain (CMSKP) is attentionally demanding, complex and multi-factorial; neuroimaging research in the population seen in pain clinics is sparse. A better understanding of the neural activity underlying attentional processes to pain related information compared to healthy controls may help inform diagnosis and management in the future.

**Methods:**

Blood oxygenation level dependent functional magnetic resonance imaging (BOLD fMRI) compared brain responses in patients with CMSKP (*n* = 15) and healthy controls (*n* = 14) while completing a modified Stroop task using pain-related, positive-emotional, and neutral control words.

**Results:**

Response times in the Stroop task were no different for CMSKP patients compared with controls, but patients were less accurate in their responses to all word types. BOLD fMRI responses during presentation of pain-related words suggested increases in neural activation in patients compared to controls in regions previously reported as being involved in pain perception and emotion: the anterior cingulate cortex, insula and primary and secondary somatosensory cortex. No fMRI differences were seen between groups in response to positive or control words.

**Conclusions:**

Using this modified Stroop tasks, specific differences were identified in brain activity between CMSKP patients and controls in response to pain-related information using fMRI. This provided evidence of differences in the way that pain-related information is processed in those with chronic complex musculoskeletal pain that were not detectable using the behavioural measures of speed and accuracy. The study may be helpful in gaining new insights into the impact of attention in those living with chronic pain.

## Background

Chronic musculoskeletal pain (CMSKP) poses a major clinical, social and economic problem [1, 2] and can be complex to manage [3]. Pain interrupts, distracts, and interferes with cognitive functioning [4] because it grasps attention [5]. Attentional bias to pain-related information can lead to mood and disability problems [6] and can constrain application of cognitively based treatments [7] and coping strategies [8].

Neuroimaging has improved our understanding of the neural processes underlying cognition, emotion and context that influence pain perception [9–11]. The majority of fMRI studies have focused on acute, experimentally-induced pain in healthy volunteers, where the subjective meaning of pain may be different in those with CMSKP [12, 13]. Relatively little is known about the neural mechanisms underlying an attentional bias in patients with CMSKP.

The Stroop paradigm focuses on the fact that cognitive interference occurs when the processing of one stimulus feature impedes the simultaneous processing of a second stimulus and is a well established paradigm for assessing attentional bias [14, 15]. It has been used in chronic pain populations to establish the degree to which patients attend to pain-related information [14, 16–18]. However not all studies show an attentional bias to pain-related and negative interference words and the specificity of effects to chronic pain (versus healthy controls) has been debated [19]. It has been proposed [20] that CMSKP overrides the interference effects in the Stroop task; pain demands attention, competing attentional demands are less important. Previous anxiety research has shown that positive words (describing a state that is desired but feared will never be achieved) provide as much interference as negative words (threatening words) and these interference effects are attributable to the extent to which the words used are related to the likely emotional concerns of patients [21]. Therefore, positive words may be useful in CMSKP studies to address previous debates.

To our knowledge, the only neuroimaging study to use a Stroop paradigm in a clinical pain population to date [22] examined patients with temporomandibular disorders matched to healthy controls. The patients had sluggish reaction times for all Stroop tasks and compared to controls, patients showed increased task-evoked responses in brain areas implicated in attention, emotional processes, motor planning and performance, and activation of the default-mode network. However, patients had mild to moderate and/or intermittent pain, and extrapolating these results to the specialist pain clinic population of CMSKP, with severe and complex pain problems, may not be appropriate.

The present study aims to examine the attentional, behavioural and activation differences between patients with complex CMSKP (i.e. those requiring specialist management in secondary care) and healthy controls using a Stroop paradigm. Using this paradigm, we will investigate whether (a) there is a general deficit in attentional control (as assessed by the modified Stroop) between patients and controls, (b) there is a specific attentional bias for pain-related stimuli (as opposed to positive emotional or neutral stimuli), (c) there are BOLD signal differences in patients compared to controls in pain and emotion related brain regions in response to the Stroop task including primary (SI) and secondary (SII) somatosensory cortices, prefrontal cortex, insula and anterior cingulate cortex (ACC) [23, 24].

## Methods

### Participants

With Dyfed Powys Research Ethics Committee approval, thirty participants were recruited and provided informed written consent for the study. Fifteen patients were recruited from a pain management program and a multidisciplinary pain clinic in South Wales and 15 matched healthy (pain-free) controls were recruited from a volunteer panel. Criteria used to match the patient with the healthy control were age, gender, educational level attainment, marital and work status. All participants received small honorarium for their participation to cover travel costs and refreshments.

Patients had been assessed by a pain specialist after primary care management and this had proven ineffective due to the complex nature of the patient’s condition. Patients had been deemed suitable for specialist pain treatment and were awaiting this treatment. Criteria for patient inclusion in the study were: a physician-diagnosis of chronic non-malignant pain (International Association for the Study of Pain, [25] and pain had to be due to osteoarthritis. Each patient had to have an average pain score of 50 and above on a numerical rating scale of 0–100 (‘No’ – ‘Worst Possible Pain’) over a three-month period prior to enrolment and to be suffering from continuous pain. Patients were only included in the study if lying supine did not specifically evoke pain and if they expected to be comfortable lying in the scanner. An additional criterion for all participants was English as their first language.

Exclusion criteria for all participants were serious metabolic, rheumatoid, vascular or diagnosed psychiatric disorders, dyslexia or unable to read written English, inability to give informed consent, contraindications to MR scanning and claustrophobia. Patients were allowed to continue on their prescribed medication as long as there had been no changes made to the dose over the preceding 3 month period.

### Questionnaires and assessment

#### Pain

Within a month prior to scanning, participants were asked about their analgesic medication and intensity of pain. Patients rated their current pain on a numerical rating scale (NRS) from 0 (no pain) to 100 (worst possible pain). Using the same scale, they also rated their worst pain, least pain, pain intensity over the last week and last 3 month period, and the degree to which the pain interfered with activities of daily living over the previous week. The 101-point (i.e. 0–100) NRS of pain intensity is recommended as a core outcome measure in clinical trials of chronic pain [26]. Prior to scanning, participants were again asked about their current pain to ensure that no significant changes had been experienced over the preceding month.

#### Psychological distress

The Hospital Depression and Anxiety Scale (HADS) [27] was used as a unidimensional measure of psychological distress [28]. HADS is a fourteen item scale, seven relating to anxiety and seven to depression. In line with the recommendation of Martin et al. [29], we adopted of a global total score of psychological distress as an alternative to the original two subscale structure in this study.

### Experimental paradigm

#### Pain-related (PR) and positive-emotional (PE) Stroop task development

The Stroop task [30] is a well-established paradigm for assessing attentional bias [14, 15]. The task used in this study was developed from the emotional counting Stroop where participants are asked to count the number of words displayed [17, 22, 24]. This paradigm is suitable for block-design fMRI studies and pain research [31, 32]. An emotional Stroop paradigm is designed with psychopathology in mind and therefore the words used as stimuli consist of items related to a particular diagnosed condition as well as more generally emotionally valenced words that are implemented as a comparison condition to reveal the disorder-specific nature of any observed Stroop effect [31]. It would be anticipated that increases in reaction times to disorder-specific versus general-emotional or neutral words would be expected to be in the patient population. Such differences would not be expected, or would be observed to a lesser extent, in healthy participants to whom the words would be less salient.

Pain-related words (affective and sensory) from the McGill Pain Questionnaire (MPQ) [33] (PRStroop) and a list of words that represented positive emotional states (e.g. ‘confident’, ‘motivated’, ‘able’) (PEStroop) were rated for salience in a pilot study (20 patients with CMSKP and 20 pain-free controls), none of whom were involved in the primary imaging study. Patients were asked to rate the words that best described their pain (affective and sensory pain words, 0 ‘does not describe my pain’, 1 ‘mildly accurate description of my pain’, 2 ‘moderately accurate description of my pain’, 3 ‘exact description of my pain’), and these were ranked from the highest scoring down to the lowest scoring across the patient group. The positive emotional words were similarly rated but by both patients and the controls (0 ‘does not describe how I feel’ to 3 ‘exact description of how I feel’) and these were scored by ranking those that scored highest for the control group and lowest for the patient group.

The decision to use positive emotional words rather than negative ones was based on the study by Mathew and Klug [21] who found that positive emotional words caused as much interference with Stroop performance in anxious patients as negative words. Given the inconsistencies in negative word use in previous Stroop studies [18], it was decided that we would examine positively valenced words in the current study. The top 16 words from each word group were used in the imaging study (see Table 1).

**Table 1:** Final word list for Stroop study

Interference block	Control block	Interference block	Control block	Interference block	Control block
Sensory Interference (Sen Inter)	Sensory Control (Sen Con)	Affective Interference (Aff Inter)	Affective Control (Aff Con)	Positive Interference (Pos Inter)	Positive Control (Pos Con)
1 aching	1 kettle	1 tiring	1 funnel	1 lively	1 fridge
2 tingling	2 armchair	2 torturing	2 saucers	2 comforted	2 lampshade
3 penetrating	3 bookshelves	3 exhausting	3 letterbox	3 liberated	3 calendars
4 hurting	4 ceiling	4 wretched	4 shelves	4 outgoing	4 cabinet
5 tender	5 plates	5 vicious	5 bucket	5 robust	5 ladder
6 pulsing	6 balcony	6 nagging	6 bedding	6 rested	6 sponge
7 stabbing	7 cupboard	7 sickening	7 polishing	7 cheerful	7 textiles
8 cramping	8 carpeted	8 agonising	8 dispenser	8 optimistic	8 appliances
9 tearing	9 laundry	9 dreadful	9 boarding	9 peaceful	9 painting
10 pressing	10 calendar	10 piercing	10 bathroom	10 enjoying	10 bedroom
11 wrenching	11 radiators	11 radiating	11 barometer	11 contented	11 bookcase
12 burning	12 glasses	12 intense	12 mirrors	12 relaxed	12 barrels
13 lacerating	13 tablecloth	13 troublesome	13 screwdriver	13 enthusiastic	13 refrigerator
14 throbbing	14 fireplace	14 miserable	14 fencing	14 achieving	14 container
15 sharp	15 chair	15 annoying	15 clothing	15 healthy	15 crystal
16 heavy	16 frame	16 killing	16 surface	16 capable	16 license

Positive emotional, sensory pain-related, and affective pain-related (collectively ‘interference’) words were then matched with neutral words (household objects) based on how often they were used in the English language, word length, and the number of orthographic neighbours (the number of words that are similar to the actual word used after changing a letter) using the English Lexical Project [34] database. Quality of matching was confirmed with statistical analysis (Mann Whitney *U* test was performed given that analyses were undertaken on a word-group level) which demonstrated no statistically significant differences between the control and interference words.

#### Imaging paradigm for PRStroop/PEStroop

**Fig. 1:**
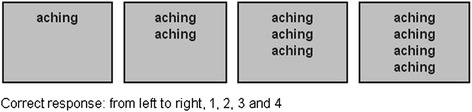
Example of 4 individual trials

The implemented protocol was based on the research by Whalen and colleagues [31]; who originally validated the emotional counting Stroop for fMRI investigations. As the original emotional paradigm was not pain specific, this led to the development of the PRStroop and PEStroop in the current study. On each trial, participants viewed sets of one to four identical words on a screen and were instructed to report the number of words displayed (see Fig. 1).

**Fig. 2:**
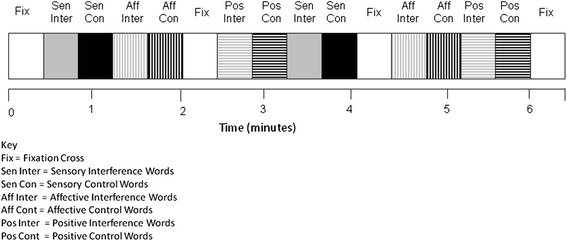
Block design for PRStroop and PEStroop task

The correct answers were always 1, 2, 3, or 4. Subjects were instructed, ‘*work as quickly as possible, but do not sacrifice accuracy for speed, and do not blur your vision in an attempt to make the task easier – keep the words in sharp focus*’. Subjects made their response using two response boxes, one held in each hand. Subjects used their middle and index finger of their left hand when their response was 1 and 2 respectively, and the index and middle finger of their right hand when their response was 3 and 4, respectively. Each trial lasted 1.5 s and there were 16 trials in a 24 s block. Each run included 16 blocks, of which there were 2 blocks for each word-type, 2 blocks for each corresponding control word set and four fixation-cross (rest) blocks (24 s duration) presented on the screen at the beginning and end of both runs and twice within a run (Fig. 2). A block consisted of one word type and the word type and appearance was randomized and counterbalanced across subjects, within runs and across runs and subjects. Subjects completed two runs of the combined PRStroop/PEStroop during MR imaging. Each run lasted 414 s so the whole session was less than 15 min, with a short break between the two runs.

### Imaging paradigm

Prior to scanning, subjects completed a 96 s practice version of the task within a realistic mock scanner. This was to familiarize subjects with the tasks and to reduce anxiety and fear for those that had not been in a scanner previously. All words used in the practice session were different to those presented in the scanning session. Responses from the training session were reviewed to ensure that the subject understood the task.

Imaging was performed on a 3 T MRI system (HDx, General Electric Healthcare, Waukesha, Wisconsin, USA) using an 8-channel receive-only head coil. Functional MRI data were acquired with a gradient-echo, echo-planar imaging sequence, scanning parameters were: repetition time (TR)/echo time (TE) = 3000 ms/35 ms, 20.5 cm field of view, acquired on a 64 x 64 matrix with 53 contiguous 3.2 mm slices. Each run consisted of 138 repetitions. For anatomic localization, a T1-weighted, three-dimensional fast-spoiled gradient echo acquisition was performed, with a voxel resolution 1x1x1 mm^3^ (scanning parameters included: TR/TE = 7.8/3 ms, 450 ms inversion time) for each participant.

### Analysis

#### Behavioural data

To test for differences in Stroop reaction times (RTs), a repeated-measures analysis of variance (RM-ANOVA) was used. The dependent variable was the RT and the fixed factor was the study group (CMSKP vs. healthy control). Run 1 and run 2 were analyzed separately to test for habituation; a comparison was undertaken between the two runs looking for statistically different response latencies. The number of accurate responses was compared between groups (CMSKP vs. healthy control) using independent t-tests. Participants were judged to be responding accurately if the number pressed on the button box corresponded to the number of words presented on the screen. Significance was set at *P*-value of less than 0.05. Statistical analysis was performed using SPSS software version 16.0 for Windows (SPSS, Chicago, Illinois, USA).

#### Image analysis

Analysis of BOLD data was performed using FEATv5.98 (FMRI Expert Analysis Tool), part of FSL (FMRIB's Software Library, www.fmrib.ox.ac.uk/fsl). The functional data for each subject was motion corrected (MCFLIRT [35]) and field maps were processed using PRELUDE + FUGUE [36, 37] to correct for field distortions in the functional data. Registration to each subject’s high resolution structural image was performed using FLIRT [35, 38] and registration to standard space was then performed using FNIRT nonlinear registration [39]. Data was smoothed spatially with a Gaussian kernel with a FWHM of 5 mm and filtered with a highpass temporal filter (cut off of 100 s) and the data was demeaned on a voxel-by-voxel basis across the time course. At the voxel level, the signal was linearly modeled (FILM-FMRIB's Improved Linear Model) with autocorrelation correction [40].

Data were analysed at three levels:

1. Data were initially analyzed at the individual subject level for each run, modelling data as the convolution of the word block with a haemodynamic response function (a gamma-variate).
2. A second-level, fixed effects analysis was performed to combine the two runs for each subject.
3. A third level, mixed effects analysis was performed to indicate differences between patients and control groups. Two third level analyses were performed, one including HADS as a covariate as suggested in a previous Stroop study [41] and one without the inclusion of HADS.

Each interference word group (sensory pain, affective pain and positive emotional) was compared with the corresponding control word group. The affective and sensory interference words were also examined when combined together to reflect the way the McGill Questionnaire is used clinically, as the word groups are not separated to provide a final score [33]. Combining of scores has been undertaken in previous Stroop research [20, 42]. For all analyses, statistic images were thresholded using clusters determined by a *Z* > 2.3 and cluster corrected (Family Wise Error) at a significance threshold of *p =* 0.05 [43]. FLAME [44] was used for the higher level analysis and examined the affective and sensory words which formed the PRStroop and positive words which formed the PEStroop. FSL was used to view the statistical parametric maps and the areas of BOLD signal differences were identified by using the Harvard-Oxford cortical and subcortical atlases.

## Results

### Demographic data and questionnaires

Twenty nine participants were scanned (5 male in the patient group, 4 in the control, 20 female, 10 in each group), age range 25 to 83 years old, including 15 patients with pain and 14 age, gender and educational level attainment-matched controls. One control subject was unable to tolerate being in the scanner and withdrew from the study. No patient complained of increased pain during the scanning period. Pain scores and HADS were compared between groups with a Mann–Whitney *U* test. As expected, patients and controls differed in pain scores and patients median current numerical rating score was 60 (range 40 – 70) (0 – ‘no pain’, 100 ‘worst possible pain’). The HADS illustrated that patients had more psychological distress compared to controls (see Table 2).

**Table 2:** Pain scores and HADS

	Patient	Control	*p* = Value
	Median values (25^th^, 75^th^ percentiles)	Median values (25^th^, 75^th^ percentiles)	Mann–Whitney test
Current pain	60 (40–70)	0 (0–0)	<0.001
0 (no pain) – 100 (worst possible pain) NRS			
Worst pain (past week)	90 (70–95)	0 (0–0)	<0.001
0 (no pain) – 100 (worst possible pain) NRS			
Least pain (past week)	35 (25–54)	0 (0–0)	<0.001
0 (no pain) – 100 (worst possible pain) NRS			
Pain intensity (past week)	64 (50–70)	0 (0–0)	<0.001
0 (no pain) – 100 (worst possible pain) NRS			
Pain intensity (average 3 months), 0 (no pain) – 100 (worst possible pain) NRS	64 (50–70)	0 (0–0)	<0.001
Pain disturbance (past week) 0 (no pain) – 100 (worst possible pain) NRS	61 (50–85)	0 (0–0)	<0.001
HADS	19 (13–23)	5 (1.5-9.75)	<0.001
<7 normal, 8–10 borderline abnormal, >11 abnormal			

Patients’ clinical characteristics are described in Table 3. Of those scanned, 2 patients and 1 control were left handed. All patients but two had previously undergone a diagnostic MRI scan and 9 volunteers had previously been scanned as participants in previous studies or for non-pain related clinical reasons. All participants reported being comfortable in the scanner.

**Table 3:** Description of the patient group

Patient	Age	Pain sites
1	29	Knees
2	59	Back, neck
3	65	Shoulders, hips
4	25	Knees, hips
5	60	Back, knees
6	61	Back, feet
7	83	Major joints
8	76	Major joints
9	65	Major joints
10	71	Back, shoulders
11	62	Back, shoulders
12	38	Back, neck
13	64	Major joints
14	56	Back, neck
15	55	Back, neck

### Behavioural responses to Stroop

There were no statistically significant RT differences for any word group (i.e., sensory, affective or positive word types, control or interference condition) between patients and controls in an individual run or combined runs (Table 4). No habituation was found; there were no differences between run 1 and run 2, and response times were not significantly different when comparing the beginning of a run with the end of the run. Comparisons between each word group and the combined group (CMSKP patients and controls) showed no Stroop effect in relation to the pain-related or positive emotional words. There were also no correlation between response times and age group; older patients did not respond significantly differently compared to the younger age groups. However, patients were significantly less accurate than controls in completing the task (Table 5). Patients were similarly inaccurate in the responses to the interference (pain and positive emotional) words as they were for control words. Level of inaccuracy was not specific to any word block or related to handedness.

**Table 4:** Response times (milliseconds). Expressed as mean (SD)

	Run 1			Run 2		
	Patients	Control	*p*-value	Patients	Control	*p*-value
Affective Control	767 (198)	713 (179)	0.11	752 (186)	688 (162)	0.031
Affective Interference	770 (194)	740 (209)	0.37	786 (179)	728 (176)	0.056
Positive Control	783 (194)	741 (181)	0.22	741 (167)	696 (175)	0.12
Positive Interference	789 (216)	704 (196)	0.015	767 (188)	698 (176)	0.040
Sensory Control	793 (198)	736 (182)	0.11	750 (177)	706 (157)	0.13
Sensory Interference	790 (226)	755 (207)	0.29	776 (192)	718 (156)	0.090

**Table 5:** Accuracy. Expressed as median (interquartile range), percentage of 16 possible correct responses

	Run 1		Run 2	
	Patients	Control	Patients	Control
Affective Control	94 % [55 % to 100 %]	100 % [94 % to 100 %]	100 % [70 % to 100 %]	100 % [94 % to 100 %]
Affective Interference	94 % [55 % to 100 %]	100 % [94 % to 100 %]	97 % [66 % to 100 %]	100 % [94 % to 100 %]
Positive Control	94 % [56 % to 100 %]	100 % [94 % to 100 %]	94 % [73 % to 100 %]	100 % [94 % to 100 %]
Positive Interference	91 % [50 % to 100 %]	97 % [88 % to 100 %]	94 % [69 % to 100 %]	100 % [94 % to 100 %]
Sensory Control	94 % [50 % to 100 %]	100 % [94 % to 100 %]	100 % [69 % to 100 %]	100 % [94 % to 100 %]
Sensory Interference	91 % [50 % to 100 %]	100 % [94 % to 100 %]	100 % [88 % to 100 %]	100 % [100 % to 100 %]

Summary data for accuracy was reported as median and interquartile range to provide some information on the asymmetry of the distribution of the data and to allow for the fixed upper limit of 100 % for accuracy as many of the participants had accuracy scores close to or at this level

Generalised linear mixed model (SPSS Version 20) was used to analyse the data. A separate analysis was carried out for each word type (Affective, Positive and Sensory) and level (Control and Interference) for both runs 1 and 2 (12 analyses in total). To allow for multiple testing, the significance level was set at 0.05/12 = 0.004. ‘Patient or Control’ and ‘repeat’ (each run comprised two repeats) were added as fixed effects and patient ID was added as a random effect, to allow for multiple responses. None of the analyses indicated a significant difference between patients and controls.

### Imaging results

There were no behavioural differences between the two runs of the Stroop task and therefore imaging analysis results were pooled across runs [32]. Whole brain analysis revealed that the interference affective pain words compared to control words showed no differences between the patients and controls.

**Fig. 3:**
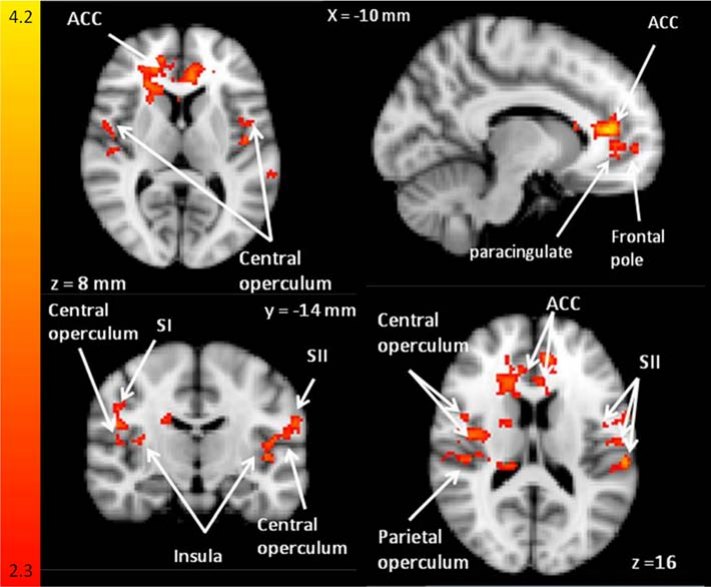
Sensory word BOLD responses. BOLD signal differences during PRStroop task comparing sensory words to the control words (patient > control groups). This z-statistic map represents these group differences in a whole brain analysis and the z-statistic map is shown in standard MNI space. The color bar shows the scale of the z-statistic (2.3 – 4.2). Cluster correction for multiple comparisons was performed at *p* < 0.05

When affective and sensory MPQ words (PRStroop) were combined in the second level analysis and in the third level analysis, differences in BOLD responses were observed in centres involved in pain, emotion and attention between pain words and control words in patients contrasted with controls when HADS was used as a covariate (see Fig. 3) and when it was not. When the third level analysis was undertaken with HADS as a covariate, 5 clusters were seen (see Table 6) and when HADS was excluded in the third level analysis, three clusters were seen (Table 7). There were no differences in BOLD responses between patients and controls to positive interference words or control words (i.e. in the PEStroop task).

**Table 6:** Group differences for the modified Stroop task during third level analysis with HADS as a covariate

	Co-ordinates			z-stat
	x	y	z	
Cluster 1 (7011 voxels, resolution of 2 mm x 2 mm x 2 mm)				
ACC (L)	−6	40	12	4.37
Caudate (R)	16	20	16	2.58
Frontal pole (L)	38	36	8	3.72
Subcallosal gyrus (L)	0	18	0	4.11
Thalamus (R)	4	−8	0	3.66
Cluster 2 (1165 voxels, resolution of 2 mm x 2 mm x 2 mm)				
Planum temporale/parietal operculum (L)	−60	−28	14	3.85
Precentral gyrus/inferior frontal/pars operculum	−58	6	28	3.35
Superior/middle temporal gyrus posterior, anterior (L)	−56	−12	−8	3.81
Supramarginal gyrus, anterior/parietal operculum (L)	−62	−28	20	4.05
Cluster 3 (526 voxels, resolution of 2 mm x 2 mm x 2 mm)				
Insula (L)	−32	−24	10	3.33
Parietal operculum (L)	−40	−28	18	3.11
Cluster 4 (493 voxels, resolution of 2 mm x 2 mm x 2 mm)				
Frontal pole (R)	28	40	40	3.23
Frontal pole and superior frontal gyrus (R)	22	38	46	3.88
Middle frontal gyrus (R)	22	28	30	3.03
Superior frontal gyrus (R)	16	28	40	3.31
Cluster 5 (394 voxels, resolution of 2 mm x 2 mm x 2 mm)				
Post central gyrus (L)	−54	−16	42	3.32
Pre/Post central gyrus (L)	−48	−14	40	3.34
Precentral gyrus (L)	−44	−8	32	3.09
Supramarginal gyrus anterior/post central gyrus (L)	−62	−28	42	3.20
Supramarginal gyrus anterior/superior (L)	−54	−38	52	3.02

**Table 7:** Group differences for the modified Stroop task during third level analysis without HADS as a covariate

	Co-ordinates			z-stat
	x	y	z	
Cluster 1 (4265 voxels, resolution of 2 mm x 2 mm x 2 mm)				
ACC (L)	−6	38	12	4.01
ACC (R)	8	22	20	3.79
ACC/paracingulate (R)	6	34	22	3.70
Caudate (R)	16	18	16	2.87
Frontal pole (R)	16	58	−8	3.90
Cluster 2 (642 voxels, resolution of 2 mm x 2 mm x 2 mm)				
Central opercular cortex (L)	−56	−14	16	3.14
Planum temporale/parietal operculum (L)	−60	−28	14	3.14
Postcentral gyrus (L)	−60	−16	24	3.31
Precentral gyrus (L)	−44	−8	32	3.28
Cluster 3 (379 voxels, resolution of 2 mm x 2 mm x 2 mm)				
Central opercular cortex (R)	50	−6	14	3.13
Central opercular cotex/Heschl’s gyrus (R)	56	−10	6	2.82
Central opercular cortex/planum temporale (R)	56	−2	6	2.91
Parietal operculum (R)	32	−24	22	3.25

**Fig. 4:**
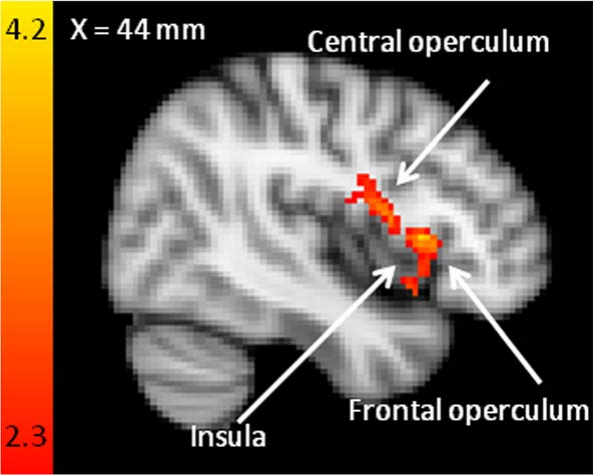
Maps comparing activation during PRStroop task. Maps comparing activation during PRStroop task contrasting sensory and affective pain words compared with control words (patients > controls). Patients with CMSKP have significantly different BOLD signal responses in sensory-discriminatory pain related regions, the affective-motivational dimension and the cognitive evaluative dimension. Each *z*-statistic map represents these group differences in a whole brain analysis. The color bar shows the scale of the *z*-statistic (2.3 – 4.2). Cluster correction for multiple comparisons was performed at *p* < 0.05

The sensory pain interference words compared to control words showed differences in BOLD signal changes in patients relative to controls in the right insular cortex, right frontal operculum and right central opercular cortex (Fig. 4) in the third level analysis.

## Discussion

To our knowledge, this is the first study that uses a Stroop paradigm in a complex CMSKP group of patients needing specialist pain management. The findings demonstrate that pain-related words used in a PRStroop task resulted in BOLD signal differences between CMSKP patients and healthy controls in pain processing centers in the brain. Larger BOLD signal increases were seen in the patient group compared to the control group in pain-related regions including the ACC, insula, parietal operculum and SI, SII (see Fig. 2). Similar activation patterns are commonly seen when physical pain stimulus in used [18]. No differences in changes in BOLD signal were seen between the patients and controls for the positive interference words. Patients were significantly less accurate in the Stroop task compared with their matched controls across all word groups.

Previous studies using pain-related versions of Stroop have been equivocal; some have not demonstrated differences in RTs [22, 41, 45] while others have found attentional bias for pain words in patients but not controls [14, 18]. Whalen et al. [31] proposed that in an emotional (but not pain-related) counting Stroop, the patient group should demonstrate RTs that are greater for interference trials than for neutral trials, whereas such a difference would not be observed in a healthy control group. They proposed that the ACC would coincide with greater response latencies and healthy participants would show a typical ‘deactivation’ in the pregenual/subgenual ventral ACC, PCC and hippocampus. In this context, our imaging results of BOLD differences in some of these regions in the absence of RT differences highlights specific differences in the processing of pain-related information that are not observable in the RT behavioural Stroop data.

The lack of a Stroop effect may imply that RTs may be an imperfect or at least less sensitive measure of cognition [46]. Patients were equally inaccurate in responding to both interference and control words in the current study, suggesting a more general impairment with cognitive performance rather than a specific attentional bias for pain-related information (i.e. information we expected to be salient and attentionally demanding in this group), and therefore this does not indicate a Stroop effect. In imaging studies of pain words using alternative paradigms to Stroop [47], changes in centers involved in pain perception have been observed, although direct comparison with our data is difficult due to use of a healthy subjects and different tasks. Nonetheless, it is clear that emotion and cognition are important in processing pain-related information. Patients were similarly inaccurate in processing the positive word category, yet there were no BOLD differences between patients and controls for this group of interference words. Therefore, we do not consider the BOLD differences to just be related to the accuracy in responding, and conclude that it appears to be the pain words that are influencing the BOLD responses in patients.

Pain has multiple dimensions; the sensory-discriminative (lateral pain pathway), affective-motivational (medial pain pathway) and cognitive-evaluative components [48]. While these three dimensions interact, it can be instructive to consider them independently to interpret these imaging results in the context of a behavioural-cognitive task. We suggest that the current study shows that in processing pain words major regions that facilitate the sensory-discriminatory component of pain can be activated in this patient population in the absence of noxious stimuli. The sensory-discriminative component involves the lateral pain pathway and the cortical areas SI and SII [23]. These two regions showed different BOLD response in patients compared to controls (see Fig. 2). SI is considered important for attentional aspects of pain processing [49] and sensory localization and intensity discrimination [50]. SII has been shown to be activated in rating pain intensity of actions depicted as words [51], and in combination with the insula (see Fig. 2), may have a role in pain discrimination [52] and the memory of pain [53]. The right caudate (see Fig. 2) is engaged during evaluation of spatial locations of noxious stimuli [54], and showed increased activation in the patient group compared with the controls during the presentation of the pain interference condition.

We also propose that pain-related words, in the absence of induced noxious stimulation, can activate the areas of the brain associated with affective-motivational aspects of pain in CMSKP patients. Regions involved in the affective-motivational dimension of pain include the insula cortex and rostral ventral ACC [55], inferior and superior parietal cortices and thalamus [49, 56–58]. This is consistent with the work of Legrain et al. [59] who proposed that the ‘pain matrix’ is largely a salience network reflecting a system involved in detecting, orienting attention towards, and reacting to the occurrence of salient sensory events. The insula receives its major input from the lateral system, but projects to the limbic system [60]. The anterior insula [61, 62] and the ACC [24, 61, 63] are associated with the evaluative-cognitive and affective-motivational aspects of pain. The insula is not only activated during painful compared to non painful touch [64, 65], but also in anticipation of pain [66], pain empathy [67] and stimulation of the insula evokes painful experiences [68]. The ACC is involved in pain affect and with the evaluation of emotional stimuli [69].

The parietal operculum and inferior parietal lobe (see Fig. 2) also showed BOLD signal differences between patients and controls. The parietal operculum is activated with pain-related images [70–72] and has a substantial role in the cortical representation of pain [73]. Combined with the inferior partietal lobe (supramarginal gyrus) it is likely to play a significant role in attention to noxious stimuli [56]. We suggest that these regions showed BOLD response differences in patients compared to controls because patients were assessing the unpleasantness associated with pain triggered by the pain words.

The cognitive-evaluative component of pain involves evaluation and interpretation of the meaning of pain and emotional distress. BOLD signal differences were seen in patients compared to controls in the central opercular cortex, paracingulate and in the left frontal pole. The central opercular cortex and frontal pole [74] are involved in memory processing and the paracingulate is involved in reality monitoring in relation to memory processing [75]. We propose the differences in these regions are related to the salience of the pain words for patients but this salience is not present in controls. The attention to pain-related words may be mediated by fear as the subcallosal cingulate cortex has a role in fear [76].

When HADS was not used as a covariate in the analysis, there appeared to be more ACC, frontal pole, central opercular cortex, Heschl’s gyrus and planum temporale weighted differences between patients and controls when compared to the third level analysis which included HADS. ACC involvement in anxiety and depression is well recognised [77–80] and a recent meta-analysis of functional MRI studies in depression noted that the superior temporal gyrus is one of the most consistently identified regions involved in the pathophysiology of depression [81]; a region which involves Heschl’s gyrus and the planum temporale. More pain-related regions were revealed between patients and controls when HADS was used as a covariate than when it was not used supporting the notion that some of the variability between subjects, driven by anxiety and depression, has been accounted for by inclusion of the HADS scores.

Nonetheless, there are a number of limitations. There are problems in studying pain-cognition interactions in patients with severe and complex chronic pain, such as seen in those referred to specialist pain centres; extricating pain-related cognitive effects from those resulting in pain treatments, especially opioids, and separating pain-related effects on cognition from the effects of the emotional distress that is a key feature of chronic pain [82]. Therefore, it has been suggested that a pragmatic approach to studying this group of patients is required [82]. Patients were not asked to stop their medications and therefore, the functional and structural changes as a result of taking these drugs over a long period [83] may have an impact on results. However, all patients had stable treatment regimens that had not been altered during the 3 months prior to imaging. It is also possible that the general increase in RT errors could be related to patients’ drug regimens and if that is correct, the pain specific results cannot be explained as drug effects. It was inappropriate to ask patients to stop their drug regimens from a clinical perspective.

## Conclusion

The use of a pain word task is non-invasive, does not require pain induction, and causes activation in brain regions associated with pain. Our study has shown that patients with complex CMSKP attend to pain-related information differently from healthy controls, which is reflected by BOLD signal changes in regions known to process pain and emotion. Patients with CMSKP did not demonstrate a specific behavioural Stroop effect, but performed worse across all Stroop tasks when compared to controls. This study adds to the literature regarding how people living with pain attend to pain-related information and offers insight to those living with complex needs where evidence is sparse. Research such as this, can support further studies looking at adapting or developing new ways of assessing cognitive biases that are more sensitive based on further imaging research to help improve diagnosis.

## Abbreviations

3 T MRI: 3 Tesla Magnetic Resonance Imaging
Aff Con: Affective Control
Aff Inter: Affective interference
ACC: Anterior cingulate cortex
BOLD fMRI: Blood oxygenation level dependent functional magnetic resonance imaging
CMSKP: Chronic musculoskeletal pain
TE: Echo time
FMRI: Functional magnetic resonance imaging
HADS: Hospital Depression and Anxiety Scale
MPQ: McGill Pain Questionnaire
Mm: Millimeters
Ms: Milliseconds
NRS: Numerical rating scale
PRStroop: Pain-related Stroop
Pos Con: Positive Control
Pos Inter: Positive Interference
PEStroop: Positive-emotional Stroop
SI: Primary somatosensory cortices
RTs: Reaction times
RM-ANOVA: Repeated-measures analysis of variance
TR: Repetition time
SII: Secondary somatosensory cortices
S: Seconds
Sen Con: Sensory control
Sen Inter: Sensory interference
USA: United States of America
